# Artificial Intelligence Applications in Pediatric Craniofacial Surgery

**DOI:** 10.3390/diagnostics15070829

**Published:** 2025-03-25

**Authors:** Lucas M. Harrison, Ragan L. Edison, Rami R. Hallac

**Affiliations:** 1 Department of Plastic Surgery, University of Texas Southwestern Medical Center, Dallas, TX 75390, USA; 2 Analytical Imaging and Modeling Center, Children’s Health Medical Center, Dallas, TX 75235, USA

**Keywords:** machine learning, craniofacial surgery, AI-assisted diagnosis, surgical planning, plastic surgery, cleft lip and palate, maxillofacial surgery, craniosynostosis, reconstructive surgery, orthognathic surgery

## Abstract

Artificial intelligence is rapidly transforming pediatric craniofacial surgery by enhancing diagnostic accuracy, improving surgical precision, and optimizing postoperative care. Machine learning and deep learning models are increasingly used to analyze complex craniofacial imaging, enabling early detection of congenital anomalies such as craniosynostosis, and cleft lip and palate. AI-driven algorithms assist in preoperative planning by identifying anatomical abnormalities, predicting surgical outcomes, and guiding personalized treatment strategies. In cleft lip and palate care, AI enhances prenatal detection, severity classification, and the design of custom therapeutic devices, while also refining speech evaluation. For craniosynostosis, AI supports automated morphology classification, severity scoring, and the assessment of surgical indications, thereby promoting diagnostic consistency and predictive outcome modeling. In orthognathic surgery, AI-driven analyses, including skeletal maturity evaluation and cephalometric assessment, inform optimal timing and diagnosis. Furthermore, in cases of craniofacial microsomia and microtia, AI improves phenotypic classification and surgical planning through precise intraoperative navigation. These advancements underscore AI’s transformative role in diagnostic accuracy, and clinical decision-making, highlighting its potential to significantly enhance evidence-based pediatric craniofacial care.

## 1. Introduction

Artificial intelligence (AI), driven by machine learning (ML) and deep learning (DL) models, is poised to transform the landscape of modern medical care. These advanced computational techniques learn from diverse datasets—including patient records, imaging studies, genetic profiles, and physiological signals—to provide actionable insights that enhance diagnostic accuracy, refine therapeutic strategies, and improve care coordination [1,2]. In clinical practice, AI has demonstrated capabilities such as robust image interpretation, precise prognostic modeling, and the identification of complex disease phenotypes, enabling data-driven decision support and personalized treatment protocols [3].

At the core of AI’s advancements in medicine are ML and DL techniques. Machine learning encompasses a range of algorithms that enable systems to learn from and make predictions based on data without being explicitly programmed for specific tasks [4,5]. Deep learning, a subset of ML, utilizes multi-layered neural networks to model complex patterns and representations in large datasets [6,7]. These technologies have proven particularly effective in handling high-dimensional data typical of medical imaging and genomics, providing superior performance in tasks such as image classification, segmentation, and anomaly detection [8,9]. The ability of DL models to autonomously extract and optimize features from raw data minimizes the need for manual feature engineering, thereby accelerating the development and deployment of AI-driven solutions in clinical settings [10,11].

Pediatric craniofacial surgery stands to benefit substantially from these technologies [12,13]. Complex anatomy, developmental factors, and the need for nuanced, patient-specific interventions require precise preoperative assessment and sophisticated three-dimensional surgical planning. AI-driven analysis can delineate anatomical landmarks, integrate multiple imaging modalities, and highlight subtle risk factors influencing perioperative decision-making [14,15]. Beyond surgical planning, real-time augmented reality (AR) guidance can align the operative field with preoperative models, enhancing intraoperative navigation and surgical accuracy. Postoperatively, large-scale data analysis can identify outcome-related patterns and refine future treatment strategies. Moreover, AI-driven patient engagement tools, including chatbots and natural language processing (NLP)-based educational platforms, can improve patient-family communication, ensuring accessible and comprehensible information tailored to individual needs [16,17].

As pediatric craniofacial procedures increasingly incorporate these technologies, the resulting synergy promises to advance both immediate and long-term patient outcomes. Enhanced predictive modeling may guide intervention timing, precise segmentation could streamline complex reconstructions, and AI-informed feedback from large postoperative datasets may accelerate technique refinement. Such innovations drive a shift toward data-centric methodologies, moving beyond conventional reliance on individual surgical expertise. This review examines the integration of AI into pediatric craniofacial surgery, underscoring its potential to shape the future of this specialized field.

This review aims to provide a comprehensive examination of how AI is shaping pediatric craniofacial surgery, with a specific focus on its applications and implications in managing cleft lip and palate, velopharyngeal insufficiency, orthognathic conditions, craniosynostosis, craniofacial microsomia, and microtia. By exploring advances in AI-driven diagnostics, surgical planning, intraoperative guidance, and postoperative assessment across these key areas, as well as examining the role of AI in surgical education, this review seeks to highlight emerging opportunities, delineate the current evidence base, and guide future research and clinical translation. In doing so, it aims to inform surgeons, researchers, and educators about the transformative potential of AI tools to enhance patient outcomes, optimize clinical workflows, and ultimately improve standards of care in pediatric craniofacial practice.

We conducted a literature review using PubMed, Scopus, and Web of Science with search terms including “artificial intelligence”, “machine learning,” “deep learning”, and key pediatric craniofacial conditions (e.g., cleft lip and palate, craniosynostosis). Inclusion criteria were peer-reviewed studies in English focusing on AI applications for diagnosis, surgical planning, intraoperative guidance, or outcome assessment in pediatric craniofacial surgery. Exclusion criteria included adult-only studies, case reports, and papers lacking technical or clinical relevance. Priority was given to studies published in the past 10 years, with exceptions for foundational research where necessary.

## 2. Cleft Lip and Palate

Cleft lip and palate are the most common congenital anomalies, affecting approximately 1 in 700 live births globally [18]. These conditions result from the incomplete fusion of tissues of the upper lip and/or the palate failing to fuse properly during embryonic development, leading to defects that can manifest as a cleft lip, cleft palate, or a combination of both, with varying degrees of severity. A cleft lip may involve only the lip or extend to include the alveolar ridge and nose, causing functional and aesthetic challenges. Similarly, a cleft palate can range from a partial opening in the soft palate to a complete separation involving both the hard and soft palates, impacting essential functions such as feeding, speech, and dental development.

The etiology of cleft lip and palate is multifactorial, with contribution from genetic predisposition and environmental factors, including maternal health, nutrition, and teratogenic exposures during pregnancy. Advances in AI have facilitated the study of genetic variants associated with orofacial clefts. For example, convolutional neural networks (CNNs) have been used to analyze single-nucleotide polymorphism (SNP) activity for predicting non-syndromic cleft lip with or without cleft palate [19]. Genetic algorithm-optimized neural networks were used to evaluate SNPs for predicting non-syndromic cleft lip with or without cleft palate in the Korean population [20].

Early detection of these congenital malformations can allow timely counseling and referral to a multidisciplinary cleft team. Management of cleft lip and palate, which requires a comprehensive, team-based approach involving plastic surgeons, orthodontists, speech therapists, and other specialists. Prenatal diagnosis, often achieved through ultrasonography, can identify these anomalies; however, the small size of fetal structures and the echogenicity of the palate bones make detection challenging [21,22]. AI-assisted techniques, such as syntactic pattern recognition and deep learning algorithms, have improved diagnostic accuracy in ultrasound imaging. A study by Jurek et al. demonstrated pattern recognition approaches for cleft palate detection to assist physicians in proper diagnosis [21]. Li et al. reported a deep learning algorithm for ultrasound-based detection with a diagnostic accuracy of 92.5% in a cohort of 632 pregnant women [22].

AI applications have expanded postnatally to improve diagnosis and classification of cleft conditions [23,24,25,26]. Kuwada et al. utilized a deep learning-based model for the identification of unilateral and bilateral cleft alveolus and palate on panoramic radiographs [23,24,25], although their clinical utility is limited due to radiation concerns. Agarwal et al. demonstrated an AI system capable of identifying cleft lip using clinical photographs, achieving an area under the receiver operating characteristic (ROC) curve of 0.95 in a dataset of 58 bilateral and 78 unilateral cleft images [26]. A study by Rosero et al. introduced an automated deep learning approach to assess lip asymmetry in patients with cleft lip, achieving a weighted accuracy of 75% in classifying asymmetry severity levels [27].

Furthermore, CNNs have been employed to classify the severity of cleft morphology. McCullough et al. used CNN-based landmark detection to grade 800 unilateral cleft lip severity, achieving a correlation coefficient of 0.892 [28]. In a study by Hayajneh et al., applied adversarial neural networks with model adaptation to assess baseline severity with high accuracy compared to human raters [29]. The generated ratings correlated closely with a score of 0.89 compared to 145 human raters.

Early intervention and tailored treatment plans are essential for optimizing functional outcomes and improving the quality of life for individuals with cleft lip and palate. Nasoalveolar molding (NAM) is a preoperative adjunct that utilizes an intraoral molding plate combined with an external nasal molding device to align the lip, alveolus, palate, and nasal segments. While highly effective, the creation of NAM devices traditionally requires significant expertise and is a time-intensive process. To address these challenges, Schiebl et al. developed an algorithm for automated generation of patient-specific NAM devices for patients with bilateral cleft lip and palate [30]. This algorithm successfully produced 3D-printable NAM devices, facilitating effective treatment for 16 patients.

Augmented Reality (AR) is an advanced technology that integrates digital information into the physical environment, often enhanced by AI-driven machine learning for improved object recognition, interaction, and adaptability. Wearable AR devices provide trainees with a unique perspective, allowing them to view surgical procedures through the eyes of the attending surgeon. These devices facilitate real-time video communication and serve as interactive reference guides during operations [31]. AR technology has demonstrated significant utility in global outreach initiatives, particularly in improving cleft care by enabling remote surgical assistance [32,33]. The virtual presence allows for longitudinal support from experienced physicians, providing guidance and reinforcing techniques acquired during surgical missions [32,33]. A 13-month education program conducted by Vyas et al., which combined augmented reality with on-site teaching, reported improved educational outcomes and enhanced patient care [33].

Alveolar bone grafting is a critical surgical procedure in the management of cleft lip and palate, typically performed during the mixed dentition phase, between 8 and 12 years of age. The procedure involves harvesting autologous bone, often from the iliac crest, and grafting it into the alveolar cleft to restore continuity of the maxillary arch. The goals of alveolar bone grafting include providing structural support for the eruption of permanent teeth, stabilizing the dental arch, and improving both facial aesthetics and functional outcomes.

Recent advancements in AI have facilitated the quantitative assessment of alveolar defects. Wang et al. developed a deep learning-based segmentation protocol to measure the length, width, and height of alveolar defects, enabling precise preoperative planning [34]. Similarly, Fujii et al. demonstrated the utility of automatic segmentation algorithms to evaluate residual grafted bone postoperatively [35]. These AI-driven tools hold significant potential for determining the volume of graft material required for surgical correction and for assessing postoperative outcomes. Miranda et al. utilized CNNs to create an automated classification system for evaluating the severity of alveolar defects based on 3D surface models of patients with cleft lip and palate, providing a robust framework for clinical decision-making [36].

The intricate nature of cleft lip repair presents significant challenges for surgical trainees due to the steep learning curve and the precision required for successful outcomes. The surgery requires mastery of delicate tissue handling and precise anatomical reconstruction, which necessitate extensive hands-on practice. CNNs based on deep learning architectures and 3D point cloud images have been developed to identify anatomical landmarks on cleft lip images [37,38,39]. These AI-driven tools assist trainees in designing surgical incisions and evaluating post-surgical outcomes, thereby enhancing the training process and improving procedural accuracy [37,38,39,40,41].

Parent and patient education are essential in the management of cleft lip and palate, particularly for ensuring optimal healing and minimizing complications after cleft palate repair. Comprehensive educational materials are essential to help parents understand the surgical process and its benefits, including improvements in feeding, speech, and overall oral health. Large language models, such as ChatGPT-4, have been employed to generate detailed and accessible educational content for caregivers [42,43,44,45,46,47,48,49]. Fazilat et al. reported high levels of satisfaction among both caregivers and surgeons with the AI-generated materials, citing their accuracy, comprehensiveness, and clarity [42]. Additionally, Lo et al. implemented AR-based educational tools, which received positive feedback for their interactive and engaging approach, further enhancing caregiver understanding and involvement in the treatment process [50]. In addition, a study assessing ChatGPT’s responses to patient inquiries on cleft lip repair demonstrated that the AI generated generally accurate and comprehensible information; however, the absence of direct citations raises concerns regarding its clinical reliability [51]. To support trainee education, Lebhar et al. explored the use of ChatGPT to generate clear and accessible surgical steps for performing cleft lip repair using the Fischer technique [52]. See Table 1.

### 2.1. Velopharyngeal Insufficiency

Velopharyngeal insufficiency (VPI) is a common functional complication in individuals with cleft lip and palate, characterized by inadequate closure of the velopharyngeal sphincter during speech. This insufficiency results in hypernasality, nasal air escape, and articulation deficits, significantly affecting speech intelligibility and communication. The etiology of VPI is multifactorial, often arising from structural anomalies or post-surgical scarring that disrupt the coordination and mobility of the soft palate and pharyngeal walls. The evaluation of VPI typically requires a multidisciplinary approach, incorporating speech assessment, nasal endoscopy, and video fluoroscopy.

Speech assessment is a vital component in the management of VPI, as speech and communication are profoundly affected by velopharyngeal dysfunction. Comprehensive speech evaluation includes the analysis of speech intelligibility, resonance, articulation, and compensatory or maladaptive speech patterns such as nasal emissions and glottal stops. Recent advancements in AI have facilitated objective speech analysis through automated processing of patient speech samples [53,54,55,56,57,58,59,60,61,62,63,64,65,66,67]. Mathad et al. developed a deep learning algorithm trained on normative speech data, demonstrating an ability to assess hypernasality with accuracy comparable to that of trained clinicians [53].

Beyond speech analysis, imaging modalities such as nasal endoscopy and video fluoroscopy are integral to diagnosing VPI by evaluating nasopharyngeal structure and function (Table 1). Ha et al. applied deep learning techniques to automate the evaluation of video fluoroscopy, achieving performance metrics comparable to those of experienced physicians [68]. Additionally, AI-driven segmentation tools have been developed to enhance the assessment of nasopharyngeal anatomy, providing more precise and reproducible measurements of velopharyngeal motion and closure patterns [69,70,71,72,73].

Management of VPI may include conservative approaches such as speech therapy or prosthetic interventions like a speech bulb or palatal lift. Surgical interventions, including pharyngeal flap surgery, sphincter pharyngoplasty, and revision palatoplasty, are often necessary for cases unresponsive to conservative measures. However, surgical correction can lead to postoperative complications, most notably obstructive sleep apnea (OSA) due to narrowing of the pharyngeal airway [74]. To address this risk, AI-based screening tools have been developed to assist in the early identification and diagnosis of OSA in patients undergoing VPI surgery [75,76,77,78,79,80,81].

AI’s integration into the assessment and management of VPI represents a significant advancement in precision medicine, offering objective diagnostic tools and predictive analytics that optimize patient outcomes. Continued research and validation of these AI-driven methodologies will further refine clinical workflows and enhance the efficacy of VPI management in individuals with cleft-related speech disorders.

### 2.2. Orthognathic Surgery

Patients with cleft lip and palate often experience maxillary hypoplasia due to intrinsic growth disturbances or surgical scarring from cleft repairs. Orthognathic surgery, typically performed after skeletal maturity, involves repositioning the maxilla, mandible, or both to correct malocclusions, improve facial symmetry, and enhance overall jaw function. Among the available techniques, the Le Fort I osteotomy is the most employed procedure for maxillary advancement, addressing midface retrusion while improving occlusion, speech, and airway function [82].

Accurate timing of orthognathic surgery is critical to achieving optimal outcomes. Kamei et al. developed an AI-driven algorithm for the detection of skeletal maturity in patients with cleft lip and palate, facilitating objective surgical timing [83]. The determination of surgical necessity is largely based on lateral cephalograms, which provide essential data on skeletal discrepancies and malocclusion severity. AI-powered algorithms have been developed to assist in the automated diagnosis and classification of patients requiring orthognathic intervention [84,85,86,87,88,89,90,91].

In patients with midfacial hypoplasia, the decision to proceed with surgical intervention depends on the severity of functional and aesthetic concerns. For individuals with minor discrepancies that do not significantly impact function, non-surgical interventions such as orthodontic treatment may suffice. Machine learning models trained on cephalometric data have been developed to provide predictive guidance on the necessity of orthognathic surgery [92,93,94,95,96,97,98]. Choi et al. applied a machine learning algorithm to a cohort of 316 patients, demonstrating a 96% accuracy in determining the need for surgery and a 91% success rate in predicting the type of surgical intervention and the necessity of extractions [97]. Additionally, early prediction models, such as those developed by Lin et al., have leveraged machine learning techniques to analyze lateral cephalograms and predict the future need for orthognathic surgery in patients as young as six years old, enabling early intervention planning [98].

Traditional orthognathic treatment protocols typically involve a preparatory phase of orthodontic decompensation to align teeth within their respective jaws before surgical repositioning. However, a surgery-first approach has gained popularity for select cases, particularly those with severe skeletal discrepancies. This approach eliminates the need for a pre-surgical orthodontic phase and can shorten overall treatment time. Chang et al. described an AI-based decision-support system that utilizes deep learning models trained on lateral cephalograms to identify candidates suitable for the surgery-first approach, thereby optimizing treatment efficiency [99].

Predicting postoperative facial appearance is a crucial aspect of surgical planning. For patients, these predictive tools provide a clear visualization of anticipated outcomes, setting realistic expectations and alleviating preoperative anxiety. Accurate predictions also enhance patient satisfaction by aligning their aesthetic goals with surgical objectives (Table 1). For surgeons, AI-assisted prediction models enhance surgical planning by ensuring that skeletal modifications translate effectively into the desired facial changes. Advanced AI algorithms have demonstrated high clinical applicability in postoperative outcome prediction [100,101,102,103,104]. Intraoperatively, augmented reality and AI-assisted custom splint fabrication have been integrated into surgical workflows to enhance precision and efficiency [105]. Additionally, AI applications in cephalometric, computed tomography, and three-dimensional imaging analyses have facilitated the objective assessment of post-treatment facial symmetry and aesthetic outcomes [106,107,108,109].

The integration of AI into orthognathic surgery has significantly improved preoperative planning, intraoperative precision, and postoperative assessment, ultimately contributing to enhanced functional and aesthetic results for patients with cleft lip and palate. Ongoing advancements in AI-driven diagnostics, predictive modeling, and intraoperative guidance continue to refine surgical decision-making and optimize treatment outcomes in this complex patient population.

## 3. Craniosynostosis

Craniosynostosis is the premature fusion of one or more cranial sutures, which normally remain open to accommodate rapid brain and skull growth. Premature suture fusion leads to abnormal cranial morphology and, in severe cases, may contribute to increased intracranial pressure, developmental delays, and neurological complications. Early diagnosis and a multidisciplinary treatment approach, often involving surgical intervention, are crucial for achieving optimal function and aesthetic outcomes.

Deformational plagiocephaly, also known as positional plagiocephaly, is a condition characterized by an asymmetrical skull flattening due to external forces on the malleable infant skull. Unlike craniosynostosis, this condition does not involve premature fusion of cranial sutures and is often managed with conservative interventions. AI-based machine learning models leveraging various imaging modalities have been developed to distinguish between craniosynostosis, deformational plagiocephaly, and normal skull morphology (Table 1) [110,111,112,113,114,115,116,117,118]. Watt et al. utilized a smartphone-based AI algorithm to diagnose deformational plagiocephaly with a sensitivity of 87.5% and specificity of 83.67% [116]. Nguyen et al. further refined AI classification methods by analyzing vertex photographs to stratify deformational plagiocephaly severity based on the Argenta classification [118]. Telehealth and AI-assisted smartphone technologies can improve access to specialized craniofacial care by facilitating remote diagnosis and appropriate referrals [116,117].

Craniosynostosis can present with varying severity depending on the affected suture, with sagittal, coronal, metopic, and lambdoid each leading to distinct cranial morphology. AI-driven diagnostic models have been developed to accurately classify these conditions [119,120,121,122,123,124,125]. Geisler et al. implemented CNNs to identify and classify non-syndromic craniosynostosis using clinical photographs, achieving an overall diagnostic accuracy of 90.6% [120].

One diagnostic challenge, particularly in metopic craniosynostosis, is differentiating true suture fusion from benign metopic ridging. To improve diagnostic precision, machine learning models have been developed to analyze three-dimensional frontal curvature data. To address this, machine learning models analyzing three-dimensional frontal curvature have been developed [126,127]. Cho et al. demonstrated that an unsupervised machine learning algorithm outperformed traditional surgeon diagnosis in distinguishing metopic craniosynostosis from benign metopic ridging, highlighting the potential of AI to enhance diagnostic accuracy and reduce subjectivity in clinical decision-making [126]. Bloch et al. demonstrated an AI-based classification system with a sensitivity of 94.4% and specificity of 92.6%, allowing for improved differentiation between pathological and benign conditions [127].

AI has also been instrumental in guiding clinical decision-making regarding surgical intervention. The severity of metopic craniosynostosis exists along a spectrum, making the decision to proceed with surgery highly subjective and surgeon-dependent. To introduce greater objectivity, AI-based severity assessment tools have been developed to quantify orbitofrontal dysmorphology. To further delineate the degree of orbitofrontal dysmorphology, objective AI tools have been developed to guide indications for operative intervention [126,127,128,129,130,131,132]. AI-generated metopic severity scores and cranial morphology deviation metrics derived from computed tomography (CT) and three-dimensional photography have demonstrated strong correlation with traditional severity indices [128,129,130,131]. The generated severity scores are comparable to previously developed severity indices and associated with aesthetic and neurocognitive outcomes [132,133,134]. Cho et al. reported a 96% concordance between an automated AI algorithm and surgeon decision-making in determining the need for surgical intervention [126].

Craniosynostosis can occur as an isolated anomaly or as part of a genetic syndrome. Unlike non-syndromic craniosynostosis, which involves isolated suture fusion, syndromic cases are part of broader conditions such as Apert, Crouzon, Pfeiffer, Saethre-Chotzen, and Muenke syndromes. These syndromes often involve mutations in genes like FGFR2, FGFR3, or TWIST1, leading to characteristic features such as midface hypoplasia, exorbitism, limb abnormalities, and, in some cases, developmental delays or hearing loss. Given the complexity of these syndromes, AI-based diagnostic frameworks have been developed to assist clinicians [135,136,137]. O’Sullivan et al. introduced a machine-learning craniofacial analysis model capable of identifying Muenke, Crouzon, and Apert syndromes with a sensitivity of 99.9% and specificity of 100%, outperforming traditional clinical diagnosis [136].

The integration of AI extends beyond diagnosis and classification to intraoperative applications. Augmented reality has emerged as a transformative tool in craniosynostosis surgery, offering enhanced visualization and precision during complex craniofacial procedures. By overlaying virtual 3D models of the patient’s anatomy onto the surgical field in real time, augmented reality enables surgeons to accurately plan and execute osteotomies and reconstructions [138,139,140,141,142,143,144]. Applications of augmented reality systems have been successfully applied in cranial vault remodeling, minimally invasive craniectomy, and orbital box osteotomy [138,139,140,141,142,143,144]. Furthermore, augmented reality technology has proven valuable in patient and caregiver education. Chen et al. found that caregivers preferred augmented reality models over two-dimensional diagrams in craniosynostosis education [145]. AI technology has also been used in the evaluation of operative outcomes in non-syndromic and syndromic patients [146,147,148,149].

## 4. Craniofacial Microsomia

Craniofacial microsomia is a congenital underdevelopment of one side of the face, primarily affecting the mandible, ear, and associated soft tissues. Advances in AI have facilitated early diagnosis and surgical planning for this condition. Baek et al. employed a convolutional neural network model to detect craniofacial microsomia from clinical photographs with high accuracy ranging from 94% to 99% [150]. Additionally, AI algorithms have been developed for cephalometric analysis and segmentation, enabling objective evaluation of preoperative malformations and postoperative outcomes. These tools provide clinicians with more precise assessments, guiding treatment planning and monitoring surgical success [151,152].

Surgical management of mandibular in craniofacial microsomia focuses on restoring facial symmetry and improving functional outcomes. Treatment options include distraction osteogenesis to gradually lengthen the underdeveloped mandible, costochondral grafting, and orthognathic to optimize jaw alignment and occlusion. Deep learning-based predictive models have been developed to estimate soft tissue profile changes following mandibular advancement surgery, allowing for more precise preoperative planning (Table 1). Ter Horst et al. demonstrated a deep-learning algorithm capable of predicting postoperative soft tissue outcomes within an acceptable error range, improving patient-specific surgical planning [153].

Mandibular osteotomies in hemifacial microsomia present unique challenges due to the inherent asymmetry and hypoplasia of the affected structures. The underdeveloped mandible often exhibits distorted anatomy, including abnormal condyles, ramus, and body, complicating osteotomies and fixation. Variability in bone quality, such as reduced cortical thickness and lower bone volume, further increases the complexity of stabilization and raises the risk of fracture or hardware failure. To address these challenges, multiple research groups have developed augmented reality platforms to assist in intraoperative navigation for mandibular osteotomies and distractor placement. These technologies enhance surgical precision by superimposing 3D models onto the operative field, providing real-time guidance [154,155,156,157,158,159,160]. Liu et al. further extended the application of augmented reality to guide facial fat grafting for soft tissue augmentation, improving volumetric restoration and symmetry [161].

Microtia, a congenital anomaly characterized by underdevelopment or absence of the external ear, frequently coexists with craniofacial microsomia. The severity of microtia varies widely, from mild structural abnormalities to complete ear absence, known as anotia. AI-driven classification models have been developed to stratify the severity of microtia based on clinical photographs. Wang et al. implemented convolutional neural networks for this purpose, enhancing diagnostic accuracy and standardizing severity assessment [162].

Surgical reconstruction of microtia typically includes reconstructive ear surgery, either using autologous cartilage or synthetic materials. Ear reconstruction presents several challenges due to the complexity of recreating a functional and aesthetically pleasing external ear. Achieving proper positioning on the side of the head is crucial for both appearance and functional outcomes, which can be challenging due to the abnormal development of the surrounding structures. Augmented reality technology has been applied to assist surgeons with placement of the ear construct [163,164,165]. Nuri et al. compared augmented reality and traditional transparent film measurements and found the difference to be within 2 mm [165]. AI tools to assess post-operative asymmetry in ear position have further been developed [166,167,168].

The integration of AI and augmented reality into the diagnosis, surgical planning, and intraoperative execution of procedures for craniofacial microsomia and microtia represents a significant advancement in craniofacial surgery. These technologies enhance diagnostic accuracy, improve preoperative assessments, and provide real-time intraoperative guidance, ultimately leading to more predictable and refined surgical outcomes. As AI and AR continue to evolve, their applications in craniofacial reconstruction will further optimize patient care and advance the precision of surgical interventions.

**Table 1:** Applications of artificial intelligence in pediatric craniofacial surgery.

Domain	AI Applications	Key Findings	References
Cleft Lip and Palate	- AI-assisted genetic analysis (CNNs for SNPs) - AI for prenatal and postnatal diagnosis (ultrasound, clinical photos) - Automated nasoalveolar molding device design - AR for surgical training and telemedicine	- Improved SNP-based prediction of cleft risk (Pearson correlation 0.50–0.83) - Enhanced ultrasound-based detection accuracy (92.5%) - CAD-generated NAM devices optimized treatment (91% success rate) - AR-supported global outreach programs enhanced training outcomes	[19,20,21,22,23,24,25,26,27,28,29,30,31,32,33]
Velopharyngeal Insufficiency (VPI)	- AI-driven speech analysis for hypernasality - AI-assisted nasopharyngeal imaging - AI-based OSA risk screening	- Deep learning models achieved clinician-level accuracy in speech assessment (r = 0.81–0.89) - AI-enhanced imaging segmentation for VPI evaluation (Dice score 0.92–0.97, detected 90% of closures) - AI models improved OSA screening before VPI surgery (Accuracy, sensitivity and specificity > 85%)	[53,54,55,56,57,58,59,60,61,62,63,64,65,66,67,68,69,70,71,72,73,74,75,76,77,78,79,80,81]
Orthognathic Surgery	- AI-driven skeletal maturity assessment - AI-assisted cephalometric analysis - AI for predicting orthognathic surgery need and outcome	- AI improved surgical timing and decision-making (Sensitivity 95.5%, specificity 95.2%, simulation error 1.1 mm) - CNN models classified skeletal discrepancies with high accuracy - AI-predicted post-surgical facial changes improved patient counseling	[82,83,84,85,86,87,88,89,90,91,92,93,94,95,96,97,98,99,100,101,102,103,104,105,106,107,108,109]
Craniosynostosis	- AI-based skull morphology classification - AI-assisted severity scoring and surgical indication - AI-powered AR for intraoperative guidance	- AI algorithms classified craniosynostosis with 90.6% accuracy - AI-based severity scores correlated with surgical decision-making (96% concordance) - AR-assisted suture mapping (error 2.4 mm)	[110,111,112,113,114,115,116,117,118,119,120,121,122,123,124,125,126,127,128,129,130,131,132,133,134,135,136,137,138,139,140,141,142,143,144,145,146,147,148,149]
Craniofacial Microsomia and Microtia	- AI-driven classification models (clinical photos, cephalometrics) - AI-assisted mandibular osteotomies and distractor placement - AR for microtia reconstruction planning	- CNN-based classification models achieved 94–99% accuracy - AR-guided osteotomies enhanced surgical precision (p < 0.05) - AR improved ear positioning accuracy within 2 mm	[150,151,152,153,154,155,156,157,158,159,160,161,162,163,164,165,166,167,168]

## 5. Limitations and Gaps

While machine learning and deep learning have demonstrated significant potential in pediatric craniofacial surgery, their widespread clinical adoption faces several challenges. Model performance often declines when applied to real-world datasets that differ from the original training data, particularly due to differences in imaging protocols, population characteristics, and regional healthcare practices. This lack of generalizability is further compounded by limited access to diverse, high-quality annotated datasets. Additionally, the cost and complexity of integrating AI tools into existing clinical workflows remain substantial barriers, especially in resource-limited settings. Regulatory approval requires extensive validation through prospective trials, which are currently lacking in many AI applications. Ethical concerns around data privacy, algorithmic bias, and transparency also require careful consideration. Addressing these challenges will be essential for ensuring that AI tools deliver consistent, equitable, and clinically meaningful benefits across diverse healthcare environments.

## 6. Future Directions

Artificial intelligence has made significant strides in healthcare, with its transformative potential particularly evident in complex surgical specialties such as pediatric craniofacial surgery. This field involves the intricate correction of congenital deformities affecting the skull, face, and jaw, requiring intricate planning, precision, and multidisciplinary collaboration. AI’s expanding role in pediatric craniofacial surgery offers promising advancements in preoperative planning, intraoperative assistance, and postoperative monitoring, ultimately improving patient outcomes and surgical efficiency.

In the preoperative phase, AI-driven technologies are poised to enhance diagnostic accuracy and streamline surgical planning. Deep learning algorithms can analyze multimodal imaging data, including computed tomography (CT), magnetic resonance imaging (MRI), and three-dimensional (3D) reconstructions, to detect subtle craniofacial abnormalities with greater precision than traditional methods. Machine learning models, trained on extensive datasets, can identify minute structural deformities, soft tissue anomalies, and developmental variations that may be imperceptible to the human eye. These insights facilitate the development of personalized surgical plans tailored to each patient’s unique anatomy, allowing surgeons to anticipate potential challenges and optimize surgical strategies.

Beyond preoperative planning, AI-driven robotic systems hold the potential to revolutionize the execution of pediatric craniofacial procedures. Given the delicate nature of craniofacial surgeries, which often involve precise bone reconstruction and meticulous soft tissue manipulation, robotic assistance could enhance surgical accuracy, reduce intraoperative complications, and improve recovery times. AI-integrated robotic platforms may also enable minimally invasive techniques, a crucial advancement for pediatric patients who are more susceptible to complications from extensive surgical interventions.

During surgery, AI can function as a real-time decision-support tool by synthesizing data from various sources, including imaging modalities, surgical instruments, and patient vitals. By continuously analyzing intraoperative parameters, AI can provide alerts regarding deviations from the planned surgical trajectory, unanticipated anatomical variations, or real-time changes in patient physiology. Additionally, AI can assist in optimizing procedural techniques, such as identifying ideal osteotomy sites, guiding implant placement, and ensuring precise cranial bone alignment—critical factors in achieving both functional and aesthetic success.

The benefits of AI extend beyond the operating room, significantly impacting postoperative monitoring and long-term patient care. AI-powered systems can track recovery trajectories, detect early signs of complications such as infections, bone displacement, or graft failure, and predict the likelihood of secondary interventions. By continuously analyzing post-surgical imaging and patient data, AI facilitates early intervention, reducing the risk of long-term morbidity.

Furthermore, AI can support longitudinal patient monitoring, particularly in pediatric populations where craniofacial structures undergo continuous growth and development. AI-driven predictive models can analyze sequential imaging data to assess craniofacial maturation, guiding clinicians on the optimal timing for additional interventions when necessary. This proactive approach ensures that developmental abnormalities are addressed promptly, minimizing the need for more extensive corrective surgeries later in life.

Techniques such as data augmentation using generative adversarial networks (GANs) and targeted handling of class imbalance have been shown to enhance model robustness and performance in medical imaging AI, including MRI-based tumor classification and other applications [169,170]. These strategies are particularly relevant when developing AI models for pediatric craniofacial surgery, where datasets are often small and highly imbalanced.

As AI technologies continue to evolve, their integration into pediatric craniofacial surgery is expected to revolutionize diagnostic workflows, surgical precision, and postoperative care. While several challenges remain, the lack of standardized, high-quality, and representative datasets presents the most significant barrier to the development and validation of AI applications in pediatric craniofacial surgery. Nonetheless, ongoing advancements in AI technology hold the potential to transform this field by enhancing diagnostic precision, surgical planning, and long-term outcome monitoring.
